# Cranial morphology reveals a lack of phylogenetic signal and rapid adaptive radiation in the bat genus *Molossus* (Chiroptera: Molossidae)

**DOI:** 10.1371/journal.pone.0320117

**Published:** 2025-04-02

**Authors:** Ana Priscila Medeiros Olímpio, Fabiano Stefanello, Beatriz Dybas da Natividade, Itiberê Piaia Bernardi, Amanda Cristiny da Silva Lima, Samira Brito Mendes, Cleison Luís da Silva Costa, Elmary da Costa Fraga, Maria Claudene Barros, Iracilda Sampaio

**Affiliations:** 1 Graduate Program in Genetics and Molecular Biology (PPGBM), Federal University of Pará (UFPA), Belém, Pará, Brazil; 2 Graduate Program in Zoology, State University of Santa Cruz (UESC), Ilhéus, Bahia, Brazil; 3 Department of Biological Sciences, University of Illinois at Chicago, Chicago, Illinois, United States of America; 4 Department of Biological Sciences, Pontifical Catholic University of Paraná (PUC-PR), Curitiba, Paraná, Brazil; 5 Graduate Program in Biodiversity and Biotechnology (Bionorte Network), Maranhão State University (UEMA), São Luís, Maranhão, Brazil; 6 Graduate Program in Biodiversity, Environment, and Health, Maranhão State University, Center for Higher Studies of Caxias, Caxias, Maranhão, Brazil; James Cook University, AUSTRALIA

## Abstract

The 16 species of *Molossus* (Chiroptera: Molossidae) are distributed throughout the Neotropical region and can be classified into two groups: one consisting of morphologically similar yet phylogenetically divergent species, and another of morphologically distinct but closely related species. This dynamic has led to frequent revisions in the systematics and taxonomy of this genus. This study aimed to analyze patterns of diversification in cranial shape and size within *Molossus* species using geometric morphometrics (GM), integrating genetic and morphological data. A total of 299 specimens from ten *Molossus* species widely distributed across the Neotropics were examined, focusing on cranial size, shape diversity, and evolution, and correlating these findings with mitochondrial DNA-based phylogenetic data. Integrated morphometric and phylogenetic analyses revealed a complex evolutionary history within *Molossus*, with most speciation events occurring during the Pleistocene, suggesting a recent rapid adaptive radiation. GM analyses demonstrated patterns of divergence in cranial size with shape conservatism, and these traits were not significantly related to phylogeny. The data indicate that phylogenetic relationships have limited influence on cranial morphology due to the lack of a strong phylogenetic signal, suggesting that ecological factors, such as diet and habitat, have played central roles in the diversification of *Molossus*.

## Introduction

Bat the genus *Molossus* É. Geoffroy, 1805 is one of the most diverse in the family Molossidae [1]. These species are distributed throughout the Neotropical region, from the southeastern United States to Argentina [2,3], including several Caribbean islands [1]. Currently, 16 species of *Molossus* are recognized.

*Molossus* species vary considerably in size and can be classified by forearm length into small (forearm <  37 mm), medium (forearm 37–43.5 mm), and large (forearm 43.5–57 mm) [4]. The genus includes a group of species that are morphologically similar but phylogenetically divergent (e.g., *M. molossus*, *M. fentoni*, *M. milleri*, and *M. verrilli*) and another group of morphologically distinct but genetically similar species (e.g., *M. aztecus*, *M. pretiosus*, *M. currentium*, and *M. sinaloae*) [3,5–8].

These characteristics have resulted in ongoing taxonomic revisions for *Molossus*, leading to frequent changes in species classification [1,2,8–11]. Such taxonomic contrasts enable the investigation of uneven diversification in morphological and behavioral traits across this genus [4].

Therefore, in this context, geometric morphometrics (GM) is a tool that can address and contribute to this taxonomic issue of *Molossus* species, as GM enables a quantitative analysis of shape, independent of size, allowing for better detection of differences among cryptic species.

GM has been successfully applied to study shape variability in relation to a range of taxonomic, evolutionary, environmental, and geographic factors in other bat groups [12,13]. Thus, this study conducted an extensive investigation to assess patterns of cranial shape and size diversification within *Molossus* through GM, integrating phylogenetic and morphological data. The following hypotheses were tested: (i) *Molossus* species exhibit distinct cranial shape and size, (ii) there is a phylogenetic signal in the cranial size and shape of these species, and (iii) evolutionary radiation influenced cranial morphological diversity in *Molossus* species. To test the hypotheses, we used geometric morphometric (GM) data to investigate whether *Molossus* species exhibit distinct cranial shape and size. We also applied phylogenetic comparative methods to analyze the influence of phylogenetic signal on the cranial size and shape of these species.

## Materials and methods

### Ethics statement

A total of 32 *Molossus molossus* specimens were gathered from Maranhão, Brazil (municipalities of Caxias, Codó, Carutapera, Godofredo Viana and Cândido Mendes). For each individual, muscle tissue samples were taken, the skull was extracted. These specimens are deposited in the Coleção de mamíferos do GENBIMOL/UEMA campus Caxias/MA, Brazil (CUMA, RRM, MRR, CESC, MAST) (Supporting information, S1 Table). The collection and handling of specimens adhered to the protocols approved by the “Committee on the Use and Care of Animals” of the American Society of Mammalogists [14] and the Ethics and Animal Experimentation Committee (CEEA) of the Veterinary Medicine Program at UEMA, under protocol no. 17/2017. Authorization for specimen collection was granted by the Ministry of the Environment (MMA) through SISBIO permits no. 42670-3 and no. 54384-1.

### Taxonomic sampling

A total, 299 specimens from ten species of *Molossus* were examined, widely distributed across the Neotropical region: *M. currentium* (2), *M. milleri* (3), *M. paranaensis* (5), *M. melini* (3), *M. fluminensis* (6), *M. aztecus* (12), *M. coibensis* (3), *M. pretiosus* (7), *M. molossus* (174), and *M. rufus* (84). The specimens are housed in the institutions listed in the (Supporting information S1 Table).

The analyzed *Molossus* species were classified by forearm length (FA) (considered a *proxy* for body size according to [4]) as follows: small (FA <  37 mm), medium (FA 37 to 43.5 mm) and large (FA 43.5 to 57 mm). Only adults, defined as having closed cranial sutures and complete epiphyseal ossification of metacarpal and phalangeal joints, were examined. Specimen identification was confirmed using taxonomic keys [1,15,16] and recent descriptions [3,17].

### GM data acquisition and statistical analyses

**Fig 1:**
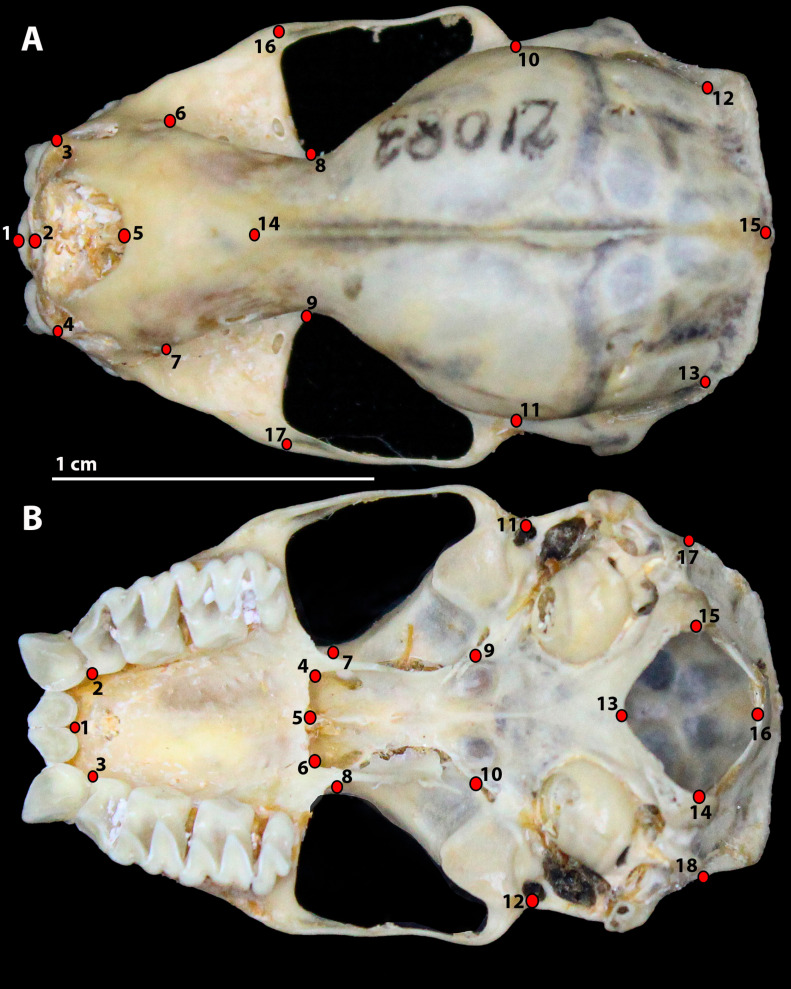
*Molossus rufus* skull showing the location of landmarks. A) dorsal view and B) ventral view. Scale =  1 centimeter.

Bidimensional images of the skulls were obtained from the ventral and dorsal views of each specimen, with a scale in centimeters. We used a digital camera (Canon T7i) with an EFS 18–55 mm lens, mounted on a photographic stand to ensure consistent angles across all images. For each skull view, type I landmarks were plotted, with 17 landmarks on the dorsal view and 18 on the ventral view (Fig 1). Landmarks were established based on linear morphometric data, given the absence of geometric morphometric (GM) studies for *Molossus* species. Additionally, we referenced anatomical landmarks established by [18] and [19] who worked with other bat groups. A TPS file (Supporting information, S2 File) was generated using the tpsUtil Software 1.83, and bidimensional coordinates of the reference points were obtained using the tpsDig 2.32 software [20].

Bidimensional coordinates were subjected to overlay using a Generalized Procrustes Analysis (GPA) which removes unwanted effects of size, position, and orientation using the function ‘procSym’ from the Morpho v.2.12 package [21] in R. A two-block partial least squares (2B-PLS) analysis was performed to assess the degree of integration between the Procrustes blocks shaping the variables, in this case, the dorsal and ventral cranial shapes. This procedure was implemented with the ‘two.b.pls’ function from the geomorph v.4.0.6 package [22]. This test allows us to determine whether parts of the bat’s skull are integrated, i.e., vary in the same way, or if there are two separate blocks of structures varying independently, possibly with different selective forces acting on each view analyzed [23]. In this case, our analyses yielded a correlation coefficient (r-PLS) of 0.7358 with p = 1e-04, indicating that the views are integrated and, therefore, only the ventral view was considered.

For geometric morphometric (GM) analyses, specimens were organized by species. We obtained Procrustes shape coordinates and a size estimator called centroid size (CS), considered a *proxy* for skull size, was determined as the square root of the sum of squared distances from each landmark to the centroid (mean of all coordinates) of the configuration [24]. Differences in centroid size among species were graphically summarized using violin plots. The number of principal components (PCs) to be analyzed was selected by measuring the correlation between the matrix of Procrustes shape distances in full shape space and pairwise Euclidean distances in reduced shape space, according to [25]. Twelve principal component analysis (PCA) axes representing over 80% of the variation in ventral shapes were then used to test cranial shape variation.

Centroid size was used as a predictor in a linear regression analysis to test for residual effects of this variable on shape. When a significant effect was observed, the residuals from this regression were used to remove residual allometry. A canonical variate analysis (CVA) with cross-validation and 9999 permutations was implemented based on PCA axes to test for cranial shape variation among species. As the effect of centroid size on shape was significant with p = 1e-04, the CVA was conducted with the shape data incorporating size effects, as well as with regression scores (i.e., residuals). These analyses were carried out using the ‘CVA’ function from the Morpho v.2.12 package [21].

First, we employed an ANOVA using species as the categorical independent variable to assess whether *Molossus* species exhibit cranial shape variation. Then, to visualize cranial shape variation, a CVA with cross-validation of 9999 permutations was performed using PCA axes. Due to the significant effect of centroid size on shape, the CVA was performed with the shape data incorporating size effects, as well as with the regression (allometry-free data). The significance level used was p =  0.05 for all statistical tests. The code used for the analyses has been deposited in our GitHub repositor https://github.com/Priscila937/Molossus_MG/tree/main.

### Molecular data

For *M. molossus*, *M. coibensis*, and *M. rufus*, total DNA was extracted from muscle tissue using the Wizard Genomic DNA Purification Kit from Promega following the manufacturer’s instructions. Sample from *Molossus coibensis* specimens housed in the mammal collection of the Federal Institute of Pará (IFPA) were included in this study and obtained through donations. The mitochondrial genes Cytochrome c oxidase subunit I (COI) and Cytochrome b (cyt b) were isolated, totaling 1,297 base pairs (COI — 617 bp, cyt b — 680 bp). The sequences generated were inspected from the chromatogram and edited in the Bioedit software [26], excluding low-quality regions when necessary, and checked for premature stop codons.

Isolation and amplification of the mitochondrial COI and cyt b genes were performed via Polymerase Chain Reaction (PCR). For the COI gene, the primers LCO 1490 and HCO 2198 described by [27] were used, following these cycling conditions: initial denaturation at 94°C for 3’, followed by 40 cycles of 94°C for 45”, annealing at 48°C for 45”, extension at 72°C for 1’30”, and a final extension at 72°C for 3’.

For the cyt b gene, primers Bat 14A and Bat 05 described by [28] were used under the following conditions: initial denaturation at 94°C for 3’, followed by 35 cycles of 94°C for 30”, annealing at 50°C for 45”, and extension at 72°C for 1’30” with a final extension at 72°C for 10’. The PCR products were purified and subjected to DNA sequencing using the Sanger method [29] on an ABI 3500 automatic DNA sequencer from Life Technologies. The new sequences generated are deposited in GenBank (Table 1).

**Table 1:** Information on *Molossus* samples for mitochondrial genes COI and cytochrome b used in genetic analyses.

Espécie	Voucher	Location	COI	Cytochrome b
*Molossus paranaensis*	MFA-ZV-M 1494 (holotype)	Argentina	OR269798	MT262819
*M. fluminensis*	non-vouchered	Argentina	OR269820	OR267006
*M. molossus*	CUMA73	Brazil, Maranhão	OP564149^*^	PQ479471^*^
*M. melini*	DZUP/CCMZ 2336	Brazil, Paraná	OR143786	PQ479472^*^
*M. coibensis*	CA28	Brazil, Pará	OR123193^*^	
	ROM 43038	Venezuela		MH058078
*M. aztecus*	CRD 2494	Mexico	MH185133	MH058047
*M. rufus*	CESC64	Brazil, Maranhão	OP538040	PQ479473^*^
*M. pretiosus*	TTU 29780	Nicaragua	MH185168	
	TTU296780			MH058079
*M. currentium*	TK 61016	Paraguay	MH185139	
	TK64016			MH058050
*M. verrilli*	ROM 125387	Dominican Republic	KX355065	
	ROM 59247			MH058057
*M. alvarezi*	ROM F49121	Mexico	MH185125	MH410726
*M. fentoni*	ROM 109176	Guyana	EF080483	
	ROM 40722			MH058051
*M. bondae*	20120811_151	Panama	MG191810	
	TK9218	Jamaica		KM387368
*M. milleri*	AMNH599033	Cuba	MH185146	
	SMNH59028			MH058056
*M. sinaloae*	ECO-SC-M3079	Mexico	MH185179	
	UNAM676			MH058094
*Promops centralis*	ROM F49126	Mexico	MH185186	
	ROM52326	Ecuador		MH058091
*Eumops auripendulus*	ROM:103342	Guyana	JF454657	
	ROM43898			MH058046

* Sequences generated in the present study.

### Molecular analyses

The molecular database consisted of 17 concatenated sequences for the two genes, and whenever possible, we included specimens with sequences available for both *loci*. In cases where this was not possible, we adopted the methodology of [3] and constructed chimeras, ensuring they were formed by individuals of the same species, country, and lineage.

Sequences were aligned using the default parameters of the ClustalW program [30]. GenBank sequences were included for the species: *M. coibensis*, *M. aztecus*, *M. pretiosus*, *M. currentium*, *M. milleri*, *M. verrilli*, *M. alvarezi*, *M. fentoni*, *M. bondae*, *M. sinaloae*, *M. fluminensis*, *M. melini*, and *M. paranaensis*. Sequences from GenBank for *Eumops auripendulus* (JF454657 and MH058046) and *Promops centralis* (MH185186 and MH058091) were included as outgroups (Table 1).

### Species tree and divergence time estimates

To reconstruct the phylogeny of *Molossus* species, we estimated the species tree and divergence times using Beast v2.7.6 [31] implemented on the CIPRES Science Gateway portal [32]. We used the relaxed molecular clock model, with parameters optimized during Markov Chain Monte Carlo (MCMC) simulations. The evolutionary model GTR with a gamma rate parameter (G) (GTR + G) was selected in the MEGA 11 program [33] as the most appropriate nucleotide substitution model for the dataset. We employed the Fossilized Birth and Death (FBD) model as the tree prior and calibrated the divergence time estimates using: I) a global mtDNA substitution rate of 0.02 (substitutions/site/Ma) [34]; II) divergence time between *Eumops* and the most recent common ancestor of *Promops* and *Molossus*, minimum of 17 Ma [35–37].

The tree was generated with MCMC simulations run for 200 million generations, sampled every 1,000 steps. Convergence and effective sample sizes (ESS >  200) were checked using the program Tracer v1.7 [38], and the first 10% was discarded as *burn-in*, generating a consensus tree from the remaining trees. Next, we annotated the combined tree files with TreeAnnotator v1.7.5 to calculate the maximum clade credibility (MCC) tree. The inferred tree was visualized in the FigTree v1.4.4 program (http://tree.bio.ed.ac.uk/software/figtree/), and nodes were considered significantly supported when posterior probabilities (PP) were above 0.95.

### Evolution of cranial shapes

To visualize the morphospaces defined by principal component analysis concerning the phylogeny of the studied species, we used the ‘physignal’ and ‘gm.prcomp’ functions from the geomorph package [39,40] implemented in R. This analysis allowed us to test the phylogenetic signal and tree configuration in shape space, termed phylomorphospace, to verify if phylogenetic relationships influence cranial shapes.

The phylogenetic tree was pruned to contain only the species analyzed here, removing taxa without GM data. For each species, we randomly selected the PC (PC1 and PC2) values of one specimen to correspond to the same number of taxa in the phylogeny.

To visualize the continuous evolution of cranial characters on the phylogenetic tree, we used the ‘contMap’ function from the phytools package [41] implemented in R. For this methodology, the mean log-transformed centroid for each species was calculated, and only species with GM data were included. For better visualization of the evolutionary scenario of cranial shape, ancestral character reconstruction was performed using the squared-change parsimony method with the fastAnc [42] function from the “ape” package in R, which estimates ancestral states for a continuous character using maximum likelihood.

## Results

### Variability of sizes and shapes in *Molossus*

**Fig 2:**
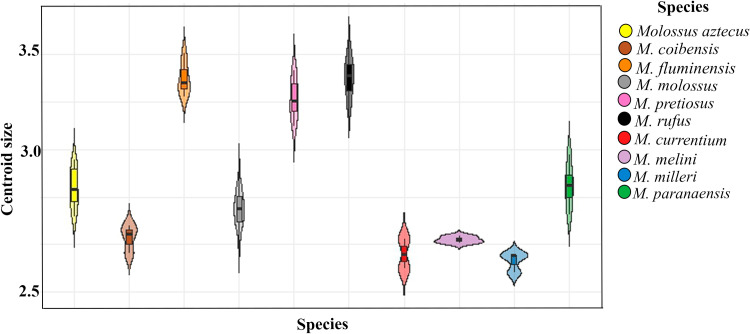
Variation in centroid size (a *proxy* for skull size) in the ventral view of the skulls of the evaluated *Molossus* species.

The skull shape varied significantly (p =  1e-04) among *Molossus* species, which showed significant variation in centroid size. These results indicate that both the size and shape of the skull vary among species. The highest centroid size values were observed in *M. rufus*, *M. pretiosus*, and *M. fluminensis*, while the lowest were found in *M. milleri* and *M. currentium* (Fig 2).

The species also differ in forearm measurements and were classified according to average forearm lengths: small (*M. milleri* [36.76 mm], *M. molossus* [35.61 mm]); medium (*M. aztecus* [39.74 mm], *M. melini* [39.93 mm], *M. coibensis* [38.86 mm]); and large (*M. currentium* [45.5 mm], *M. paranaensis* [49.95 mm], *M. fluminensis* [50.66 mm], *M. rufus* [47.85 mm], *M. pretiosus* [48.24 mm]).

**Fig 3:**
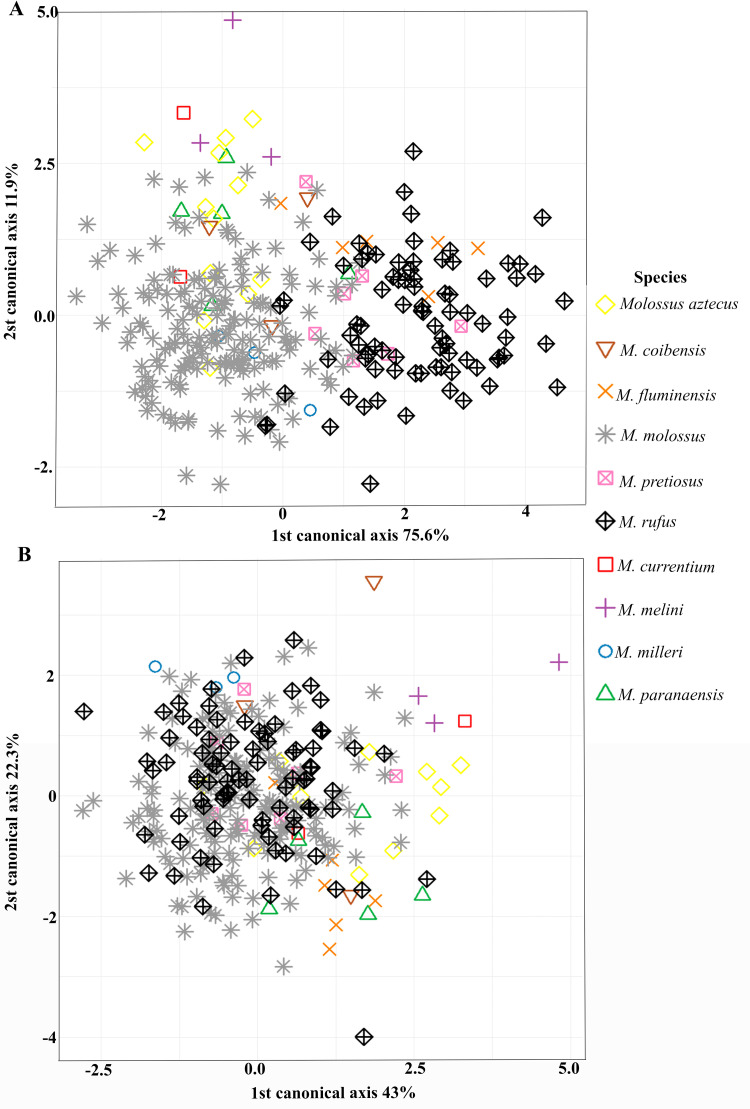
A) Canonical Variate Analysis (CVA) for cranial shape data in the ventral view of *Molossus* species; and B) CVA with regression scores (residuals).

The CVA analyses using cranial shape data from the ventral view showed a correct classification rate of 79% after cross-validation, revealing the separation between large-sized *Molossus* species (*M. rufus*, *M. fluminensis*, and *M. pretiosus*) and small to medium-sized species (*M. currentium*, *M. milleri*, *M. paranaensis*, *M. melini*, *M. aztecus*, *M. coibensis*, and *M. molossus*). An overlap was also observed between the morphologically distinct species *M. molossus* and *M. rufus*, which have small and large body sizes respectively (Fig 3A).

However, CVAs with cranial shape residuals (free from residual allometry) did not show discrimination between species. In this scenario, morphologically distinct species of different body sizes overlapped, indicating that *Molossus* species exhibit a certain degree of cranial shape conservatism, with a correct classification rate of 56% after cross-validation (Fig 3B).

**Fig 4:**
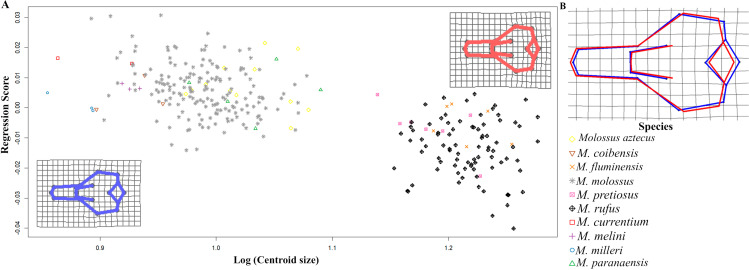
A) Regression analysis of cranial shape scores and log centroid size of *Molossus* species, showing two clusters: large-sized specimens (right) and small-sized specimens (left). The deformation grids represent shape variation associated with centroid size. Cranial shape of large species is shown in red and small to medium-sized specimens in blue. B) Cranial shape overlap.

Additionally, the effect of centroid size on cranial shape was notable, suggesting that species exhibit different cranial shapes associated with relative skull size. Small *Molossus* species displayed shorter rostra with longer cranial vaults, whereas large species had longer rostra and shorter, wider cranial vaults (Fig 4).

**Fig 5:**
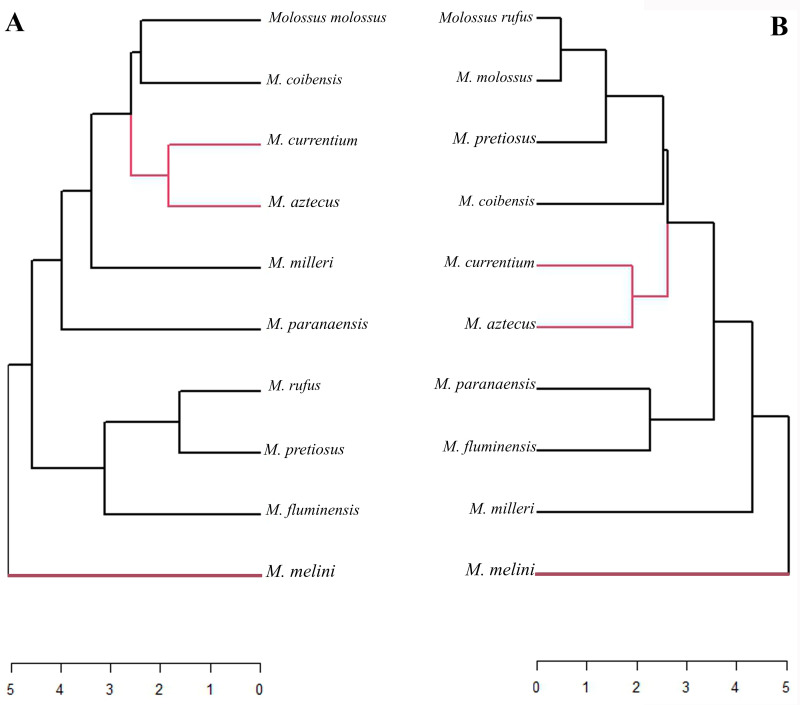
Mahalanobis distance of cranial morphology from the ventral view of *Molossus* species. A) Shape data scores. B) Regression scores (residuals). The red branches represent species that clustered together in both analyses.

The results of the Mahalanobis distance considering shape data under residual allometry effects (Fig 5A) showed consistent results with the formation of two clusters: the first includes the large species, which differentiate from the others, and the second group includes the medium and small-sized species.

When we performed this analysis considering only the regression scores, free from residual size effects, this separation was not observed, and species like *M. rufus* and *M. molossus* appeared more similar, as well as *M. fluminensis* and *M. paranaensis*. *M. currentium* and *M. aztecus* were similar in cranial shape in both analyses (Fig 5B). *M. melini* was distinct from the other species in both analyses.

### Phylogeny and phylogenetic signal

**Fig 6:**
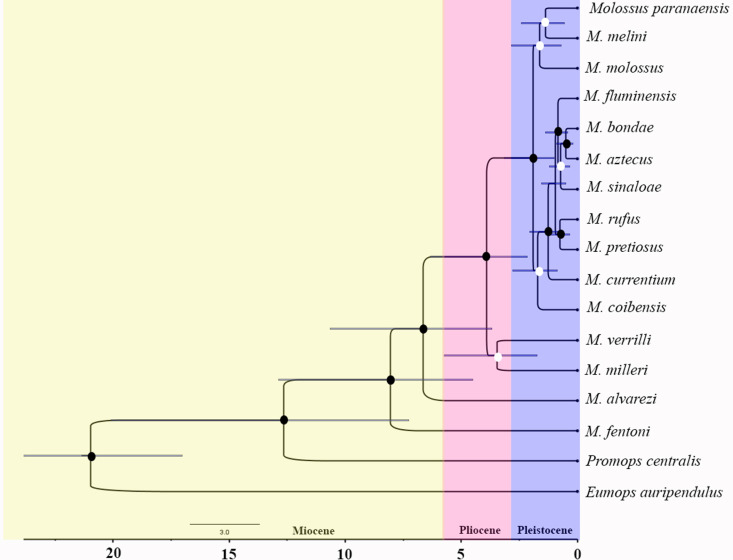
Bayesian tree and divergence times of *Molossus* species inferred from mitochondrial gene sequences COI and cytochrome b. The horizontal blue bars represent the 95% HPD intervals for branch lengths, and the circles at the nodes represent posterior probability values. White circles indicate pp values ranging from 0.54 to 0.79, while black circles indicate pp values >  0.8.

The diversification time of *Molossus* species was estimated between 12.5 million years ago (late Miocene – early Pliocene) (Fig 6). The species *M. fentoni* was recovered as the sister group (the most basal) of the remaining species in the genus. The second cladogenetic event corresponded to the divergence of *M. alvarezi* from the other species. *M. milleri* and *M. verrilli* were recovered as sister groups, with low branch support, with divergence estimated during the Pliocene. Most diversification events in the genus occurred in the Pleistocene, with most species emerging around 2.5 million years ago. For these species, it is observed that *M. fluminensis*, *M. bondae*, *M. aztecus*, *M. sinaloae*, *M. rufus*, *M. pretiosus*, *M. currentium* cluster in a strongly supported clade, while *M. molossus*, *M. paranaensis*, and *M. melini* form a clade with low support.

**Fig 7:**
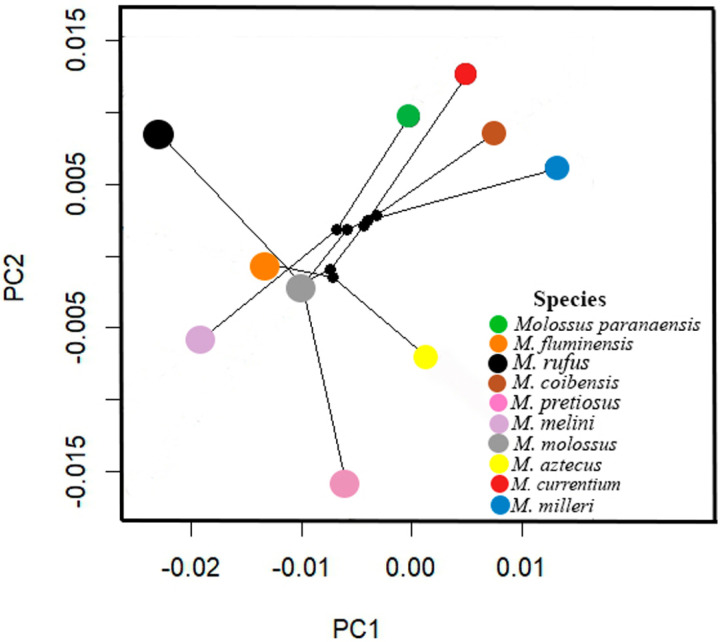
Phylomorphospace based on the cranial shape from the ventral view of the analyzed *Molossus* species.

In the phylomorphospace analyses, the PC1 axis explains 89.7% of the total variation, with no significant phylogenetic signal observed in the ventral cranial shape variation (k =  0.613 and p =  0.247). Phylogenetically close species occupy different morphospaces (Fig 7).

**Fig 8:**
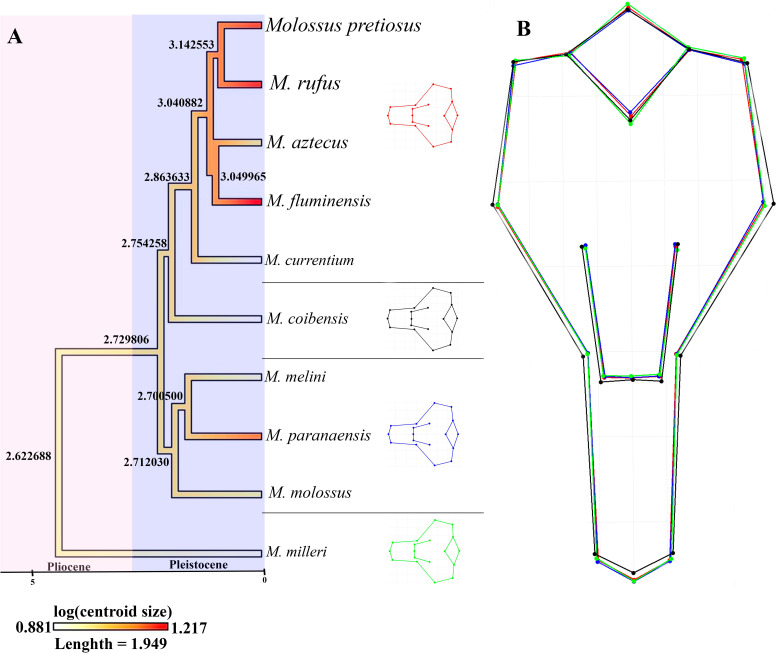
A) Changes in skull size associated with phylogenetic events of *Molossus* species; B) Cranial shape overlap. The values at the internal nodes of the phylogenetic tree correspond to estimates of ancestral centroid sizes.

When analyzing the evolution of skull size in *Molossus* species (Fig 8) associated with phylogenetic reconstruction, a variation in centroid sizes was observed with a tendency toward increased size along the evolutionary clade of *M. rufus*, *M. pretiosus*, and *M. fluminensis*. These results may be related to evolutionary adaptations to different environments or selective pressures. Given these results, the variability in observed shapes provides information about the magnitude and direction of evolutionary change.

## Discussion

### Diversification of skull shape and size in *Molossus*

This study analyzed the diversification patterns of skull shape and size in *Molossus* species using geometric morphometrics, integrated with phylogenetic and evolutionary data. Our results reveal the rich evolutionary history of the genus, with *Molossus* species exhibiting patterns of skull size divergence and shape conservatism, alongside evidence of recent radiation. Previous studies have highlighted the challenges in morphologically distinguishing species within the genus due to low levels of morphological differentiation and a tendency towards genetic conservatism [2,3,37,43].

Regarding size, *Molossus* species differ both in skull and forearm measurements, categorizing them into large, medium, and small sizes. *M. melini* have medium body size with small skull, while *M. paranaensis* is a large species with a medium-sized skull. These variations in body proportions may be closely related to the ecological and evolutionary contexts of the species [44,45].

In terms of skull shape, the analyzed species showed conserved cranial shapes, without significant differentiation among them, especially when the residual effect of centroid size is removed. Similar results were obtained by [13] when analyzing the cranial shape of species of *Artibeus* (Chiroptera, Phyllostomidae) (Olfers, 1818), which did not show differentiation between *A. cinereus* (Leach, 1821) and other large species of the genus, such as *A. lituratus* (Olfers, 1818). These species presented a skull with a longer rostrum and shorter, wider cranial vault, a pattern also observed in large *Molossus* species. According to [46], both cranial morphology and function are under strong selective pressure, being adjusted by behaviors and dietary characteristics, facilitating food acquisition and processing.

[47] evaluated the skull and dentition shapes of different bat families and observed substantial overlap in dentition shape between phyllostomids and other clades, but limited overlap in skull shape. They suggest this occurs because dentition mainly functions in food processing, while the skull serves multiple functions, imposing more constraints on its evolution as observed in *Molossus*, which displayed a more conserved skull shape. This characteristic is due to the skull’s role in housing the brain and sensory organs such as hearing, vision, and smell [48–50].

Small *Molossus* species displayed shorter rostra with longer cranial vaults, while large species had longer rostra and shorter, wider cranial vaults. [51] points out that cranial morphology varies concerning food hardness; insectivorous bats that feed on hard-shelled insects have short skulls and thick jaws, while those consuming soft-shelled insects have long, delicate jaws. [52] states that skull size can also vary according to prey size, asserting that bats feeding on larger prey have bigger heads, more robust dentition, and larger mouth openings compared to those consuming smaller prey.

[53] and [52] recorded for *M. molossus*, *M. rufus*, and *M. pretiosus* the ingestion of both hard and soft items, including representatives of different insect orders such as Coleoptera, Lepidoptera, Hymenoptera, Odonata, Orthoptera, Hemiptera, Diptera, and Hymenoptera. This dietary variation suggests that cranial morphology in *Molossus* may reflect selective pressures imposed by their diverse feeding strategies, indicating functional adaptation favoring efficiency in exploiting different food resources available in their environment. *Molossus* species are exclusively insectivorous, but there are few studies detailing their dietary preferences.

### Phylogenetic implications

In the phylomorphospace, it was observed that phylogenetically close species of *Molossus* exhibit divergent phenotypes, with branches originating from a common ancestor and separating in distinct directions, while converging with branches from other parts of the morphospace. [54] emphasize that phenotypic variables associated with a phylogeny and statistical analyses can provide insights into morphological evolution and the pace of changes within specific groups, such as bats. The absence of phylogenetic signal in the cranial shape variation of *Molossus* reinforces the hypothesis that morphological adaptations may have been guided more by ecological factors than by direct evolutionary relationships. [55] suggest that the emergence of cranial specializations, both morphological and functional, may be associated with a diversity of feeding habits, different types of echolocation emissions, and varied foraging strategies.

[4], consistent with our findings, did not find a correlation between phylogeny and morphological characters, specifically regarding the shape of the occipital complex, the shape of the infraorbital foramen, and the development of the sagittal crest. This absence of phylogenetic signal has also been observed in studies of cranial shape in the works of [56] with xenarthrans, [57] with hominids, and [13] with bats. However, for *Molossus*, [4] observed a low positive correlation between morphological characters, such as fur pattern, forearm length, and dentition, and phylogenetic distances, suggesting stability of these character states in the phylogeny, supporting the hypothesis of morphological stasis occurring in certain clades within *Molossus*. According to these authors, such results help explain why similar species of *Molossus* do not always form monophyletic groups.

When associating phylogeny with the evolution of skull size, it is observed that there is a trend toward an increase throughout the evolution of the species *M. rufus*, *M. pretiosus*, and *M. fluminensis*. Our data for the group’s phylogeny indicate a relatively recent radiation in *Molossus* during the Miocene, approximately 7 million years ago, while [4] proposed that the first cladogenetic event within *Molossus* occurred 6 million years ago with the divergence of *M. fentoni* from other *Molossus* species. Similar to our results, it shows that the majority of speciation events occurred in the Pleistocene.

The phylogenetic pattern obtained with the mitochondrial genes COI and cyt b recovered the monophyly of the genus and was consistent with previous studies by [3] and [1]. However, some divergences were observed, such as in the case of *M. sinaloae*, which grouped differently compared to previous results, suggesting that new molecular data may continue to refine our understanding of evolutionary relationships within *Molossus*. In summary, our findings not only broaden our understanding of the evolution of skull size and shape in *Molossus* but also highlight the importance of considering both phylogenetic history and current ecological pressures when studying morphological diversity in clades with wide geographical and ecological distribution.

## Conclusion

The results of this study highlight the complexity of morphological evolution in the genus *Molossus*. The coexistence of cranial form conservatism with significant divergences in size suggests that ecological factors, such as diets and habitat, play central roles in the diversification of these species. Regarding skull and forearm size, there is a clear distinction between large, small, and medium-sized species. Larger species, such as *M. rufus*, *M. pretiosus*, and *M. fluminensis*, exhibited a shorter and wider neurocranium with a longer rostrum, whereas smaller species presented a shorter rostrum with a longer neurocranium. When comparing small and large species, we found a strong effect of residual allometry influencing shape, supporting the conservatism hypothesis for cranial form without differentiation among morphologically distinct species like *M. molossus* and *M. rufus*.

The phylogenetic analysis indicated that the diversification of *Molossus* species primarily occurred during the Pleistocene, with most speciation events occurring approximately 2.5 million years ago. These data, along with MG analysis, did not reveal a significant correlation between phylogenetic proximity and morphological similarity, suggesting that the evolution of cranial shape is not influenced by phylogenetic relationships. This dissociation may result from selective pressures or differentiated ecological adaptations, as well as the explosive diversification pattern that led to the species we know today.

These findings not only broaden our understanding of morphological evolution in *Molossus* but also raise new questions about the selective pressures that shape phenotypic characteristics in different environmental contexts. These discoveries contribute to our comprehension of morphological evolution in *Molossus* and underscore the importance of integrative studies to unravel the evolutionary and ecological patterns of the species.

## Supporting information

S1 Table Specimens of *Molossus* included in the morphogeometric analyses. (DOCX)

S2 File A TPS file with the coordinates of the landmarks. (TXT)
