# Supplementary material for: Cranial morphology reveals a lack of phylogenetic signal and rapid adaptive radiation in the bat genus Molossus (Chiroptera: Molossidae)
Source: PLoS One. 2025 Apr 2;20(4):e0320117. doi: 10.1371/journal.pone.0320117 (PMC11964253; doi:10.1371/journal.pone.0320117)
Supplement: S1 Table — (DOCX) [file pone.0320117.s001.docx]

**Supporting information**

The specimens are housed in the following institutions: Colección Mamíferos Lillo, Universidad Nacional de Tucumán, Argentina (CML); Colección de Vertebrados of the Instituto Nacional de Limnología (INALI), Argentina; Colección de Mamíferos of the Museo Provincial de Ciencias Naturales - Florentino Ameghino (MFA-ZV-M); Florida Museum of Natural History, USA (FLMNH); Coleção de Mamíferos da Universidade Federal de Lavras, Brazil (CMUFLA); Coleção de Mamíferos do museu de História Natural do Ceará Professor Dias da Rocha (MHNCE/UECE); Museu de Zoologia da Universidade de São Paulo, Brazil (MZUSP); Coleção de mamíferos da Universidade Federal de Pernambuco, Brazil (UFPE, DG); Universidade Federal da Paraíba, Brazil (UFPB); Coleção Científica de Mastozoologia, Universidade Federal do Paraná, Brazil (DZUP/CCMZ); Coleção de mamíferos do Museu de Zoologia da Pontifícia Universidade Católica do Paraná (PUCPR); Coleção de mamíferos do GENBIMOL/UEMA (CUMA, RRM, MRR, CESC, MAST)”.

**S1 Table.** Specimens of *Molossus* included in the morphogeometric analyses.

| **Museum/ Código** | | **Species** | **Country** | **City** |
| --- | --- | --- | --- | --- |
| **1** | CML1816 | *M. currentium* | Argentina | Formosa, Pirané, El Colorado |
| **2** | CML1817 | *M. currentium* | Argentina | Formosa, Pirané, El Colorado |
| **3** | FLMNH 23821 | *M. milleri* | Jamaica | St. Elizabeth |
| **4** | FLMNH 23820 | *M. milleri* | Jamaica | St. Elizabeth |
| **5** | FLMNH 23822 | *M. milleri* | Jamaica | St. Elizabeth |
| **6** | INALI A390 | *M. paranaensis* | Argentina | San Jerónimo, Santa Fe province |
| **7** | INALI A457 | *M. paranaensis* | Argentina | Estancia Las Gamas, Vera, Santa Fe province |
| **8** | INALI A588 | *M. paranaensis* | Argentina | Establecimiento Inchala, La Picada, Paraná, Entre Ríos province |
| **9** | INALI A589 | *M. paranaensis* | Argentina | Establecimiento Inchala, La Picada, Paraná, Entre Ríos province |
| **10** | MFA-ZV-M 1494 | *M. paranaensis* | Argentina | Sociedad Rural “Las Colonias”, Esperanza, Santa Fe province |
| **11** | DZUP/CCMZ 2337 | *M. melini* | Brazil | Curitiba, Paraná |
| **12** | DZUP/CCMZ 2338 | *M. melini* | Brazil | Curitiba, Paraná |
| **13** | DZUP/CCMZ 2339 | *M. melini* | Brazil | Curitiba, Paraná |
| **14** | INALI A115 | *M. fluminensis* | Argentina | Corrientes, Concepción, Estancia |
| **15** | INALI A315 | *M. fluminensis* | Argentina | Santa Fe, La Capital, Santa Fe city |
| **16** | INALI A512 | *M. fluminensis* | Argentina | General Obligado, Villa Ocampo, Portal del Humedal |
| **17** | INALI A513 | *M. fluminensis* | Argentina | General Obligado, Villa Ocampo, Portal del Humedal |
| **18** | INALI A694 | *M. fluminensis* | Argentina | Villa Ocampo, Puerto Ocampo |
| **19** | INALI A114 | *M. fluminensis* | Argentina | Corrientes, Concepción, Estancia |
| **20** | MZUSP_16841 | *M. aztecus* | Brazil | Crato, Ceará |
| **21** | MZUSP_16814 | *M. aztecus* | Brazil | Exu, Pernambuco |
| **22** | MZUSP_10269 | *M. aztecus* | Brazil | Ilha Bela, São Paulo |
| **23** | MZUSP_16861 | *M. aztecus* | Brazil | Crato, Ceará |
| **24** | MZUSP_16800 | *M. aztecus* | Brazil | Exu, Pernambuco |
| **25** | MZUSP_16834 | *M. aztecus* | Brazil | Crato, Ceará |
| **26** | DZUP/CCMZ2183 | *M. aztecus* | Brazil | Ratones, Florianopólis |
| **27** | CMUFLA 356 | *M. aztecus* | Brazil | Lavras, Minas Gerais |
| **28** | CMUFLA 399 | *M. aztecus* | Brazil | Lavras, Minas Gerais |
| **29** | CMUFLA 414 | *M. aztecus* | Brazil | Lavras, Minas Gerais |
| **30** | CMUFLA 415 | *M. aztecus* | Brazil | Lavras, Minas Gerais |
| **31** | CMUFLA 416 | *M. aztecus* | Brazil | Lavras, Minas Gerais |
| **32** | MZUSP_28782 | *M. coibensis* | Brazil | Brasilândia, Mato Grosso do Sul |
| **33** | MZUSP_28689 | *M. coibensis* | Brazil | Brasilândia, Mato Grosso do Sul |
| **34** | MZUSP_2672 | *M. coibensis* | Brazil | Cidade da Barra, Bahia |
| **35** | MZUSP_36607 | *M. pretiosus* | Brazil | Santana do Araguaia, Pará |
| **36** | MZUSP_36603 | *M. pretiosus* | Brazil | Santana do Araguaia, Pará |
| **37** | MZUSP_36606 | *M. pretiosus* | Brazil | Santana do Araguaia, Pará |
| **38** | MZUSP_36604 | *M. pretiosus* | Brazil | Santana do Araguaia, Pará |
| **39** | MZUSP_36605 | *M. pretiosus* | Brazil | Santana do Araguaia, Pará |
| **40** | DZUP/CCMZ792 | *M. pretiosus* | Brazil | Parque estadual do Turvo, Rio Grande do Sul |
| **41** | MHNCE-MAM00218 | *M. pretiosus* | Brazil | Pacoti, Ceará |
| **42** | UFPB 1656 | *M. molossus* | Brazil | Faz. D'aguia, Praia de Costinha, Paraíba |
| **43** | UFPB 1657 | *M. molossus* | Brazil | Faz. D'aguia, Praia de Costinha, Paraíba |
| **44** | UFPB 1648 | *M. molossus* | Brazil | Faz. D'aguia, Praia de Costinha, Paraíba |
| **45** | UFPE 1367 | *M. molossus* | Brazil | Igaraçu, Pernambuco |
| **46** | UFPE 599 | *M. molossus* | Brazil | Mamanguape, Paraíba |
| **47** | UFPB 1654 | *M. molossus* | Brazil | Campus universitário UFPB, João Pessoa |
| **48** | UFPB 4114 | *M. molossus* | Brazil | APA da Barra do rio Mamamguape, Paraíba |
| **49** | UFPB 1653 | *M. molossus* | Brazil | Rancho mineiro, São Lourenço da Mata, Pernambuco |
| **50** | UFPB 1642 | *M. molossus* | Brazil | Mata do Buraquinho, João Pessoa, Paraíba |
| **51** | UFPE 2174 | *M. molossus* | Brazil | Igaraçu, Pernambuco |
| **52** | DZUP/CCMZ961 | *M. molossus* | Brazil | Associação de Artesãos, Guaraqueçaba |
| **53** | DZUP/CCMZ1779 | *M. molossus* | Brazil | Santa Catarina |
| **54** | PUCPR2265 | *M. molossus* | Brazil | Tijucas do Sul, Paraná |
| **55** | MZUSP_31593 | *M. molossus* | Brazil | Cajuru, São Paulo |
| **56** | MZUSP_31591 | *M. molossus* | Brazil | Cajuru, São Paulo |
| **57** | MZUSP_31848 | *M. molossus* | Brazil | Ubatuba, São Paulo |
| **58** | MZUSP_21093 | *M. molossus* | Brazil | São Paulo |
| **59** | MZUSP_10273 | *M. molossus* | Brazil | Ilha Bela, São Paulo |
| **60** | MZUSP_10272 | *M. molossus* | Brazil | Ilha Bela, São Paulo |
| **61** | MZUSP_10268 | *M. molossus* | Brazil | Ilha Bela, São Paulo |
| **62** | MZUSP_29455 | *M. molossus* | Brazil | Ubatuba, São Paulo |
| **63** | MZUSP_29454 | *M. molossus* | Brazil | Ubatuba, São Paulo |
| **64** | DZUP/CCMZ1216 | *M. molossus* | Brazil | RPPN Monte sinai, Maua da Serra, Paraná |
| **65** | DZUP/CCMZ2309 | *M. molossus* | Brazil | Guarapuava, Paraná |
| **66** | UFPE 600 | *M. molossus* | Brazil | Mamanguape, Paraíba |
| **67** | UFPE 2220 | *M. molossus* | Brazil | Rio Formoso, Pernambuco |
| **68** | UFPE 2169 | *M. molossus* | Brazil | Itamaracá, Pernambuco |
| **69** | UFPB 1647 | *M. molossus* | Brazil | Mata do Buraquinho, João Pessoa |
| **70** | UFPB 583 | *M. molossus* | Brazil | Estação ecológica de pau Brasil, 15KM NW de Porto Seguro, Bahia |
| **71** | UFPB 1658 | *M. molossus* | Brazil | Rancho mineiro, São Lourenço da Mata, Pernambuco |
| **72** | UFPB 1645 | *M. molossus* | Brazil | Mata do Buraquinho, João Pessoa, Paraíba |
| **73** | UFPB 1663 | *M. molossus* | Brazil | Mata do Buraquinho, João Pessoa, Paraíba |
| **74** | UFPB 1659 | *M. molossus* | Brazil | Mata do Buraquinho, João Pessoa, Paraíba |
| **75** | UFPB 1650 | *M. molossus* | Brazil | Conj. Castelo branco III, Paraíba |
| **76** | MZUSP_386 | *M. molossus* | Argentina | Goya; Argentina, Provincia de Corrientes, Departamento de Goya |
| **77** | CUMA 34 | *M. molossus* | Brazil | Caxias, Maranhão |
| **78** | MZUSP_1365 | *M. molossus* | Brazil | Barra, Bahia |
| **79** | MZUSP_17577 | *M. molossus* | Brazil | Ribeirão Preto, São Paulo |
| **80** | CUMA 32 | *M. molossus* | Brazil | Caxias, Maranhão |
| **81** | CUMA 53 | *M. molossus* | Brazil | Codó, Maranhão |
| **82** | CUMA 57 | *M. molossus* | Brazil | Codó, Maranhão |
| **83** | CUMA 73 | *M. molossus* | Brazil | Codó, Maranhão |
| **84** | CUMA 75 | *M. molossus* | Brazil | Codó, Maranhão |
| **85** | MZUSP_16845 | *M. molossus* | Brazil | Crato, Ceará |
| **86** | MZUSP_16830 | *M. molossus* | Brazil | Crato, Ceará |
| **87** | MZUSP_16853 | *M. molossus* | Brazil | Crato, Ceará |
| **88** | MZUSP_16843 | *M. molossus* | Brazil | Crato, Ceará |
| **89** | MZUSP_16842 | *M. molossus* | Brazil | Crato, Ceará |
| **90** | MZUSP_16840 | *M. molossus* | Brazil | Crato, Ceará |
| **91** | MZUSP_16852 | *M. molossus* | Brazil | Crato, Ceará |
| **92** | MZUSP_16838 | *M. molossus* | Brazil | Crato, Ceará |
| **93** | MZUSP_16839 | *M. molossus* | Brazil | Crato, Ceará |
| **94** | MZUSP_16844 | *M. molossus* | Brazil | Crato, Ceará |
| **95** | MZUSP_16831 | *M. molossus* | Brazil | Crato, Ceará |
| **96** | MZUSP_16808 | *M. molossus* | Brazil | Crato, Ceará |
| **97** | MZUSP_16794 | *M. molossus* | Brazil | Exu, Pernambuco |
| **98** | MZUSP_16799 | *M. molossus* | Brazil | Exu, Pernambuco |
| **99** | MZUSP_16822 | *M. molossus* | Brazil | Exu, Pernambuco |
| **100** | MZUSP_16824 | *M. molossus* | Brazil | Exu, Pernambuco |
| **101** | MZUSP_16801 | *M. molossus* | Brazil | Exu, Pernambuco |
| **102** | MZUSP_16793 | *M. molossus* | Brazil | Exu, Pernambuco |
| **103** | MZUSP_16795 | *M. molossus* | Brazil | Exu, Pernambuco |
| **104** | MZUSP_16820 | *M. molossus* | Brazil | Exu, Pernambuco |
| **105** | MZUSP_16823 | *M. molossus* | Brazil | Exu, Pernambuco |
| **106** | MZUSP_16806 | *M. molossus* | Brazil | Exu, Pernambuco |
| **107** | MZUSP_16825 | *M. molossus* | Brazil | Exu, Pernambuco |
| **108** | MZUSP_16819 | *M. molossus* | Brazil | Exu, Pernambuco |
| **109** | MZUSP_16864 | *M. molossus* | Brazil | Valença do Piauí, Piauí |
| **110** | MZUSP_16865 | *M. molossus* | Brazil | Valença do Piauí, Piauí |
| **111** | MZUSP_16863 | *M. molossus* | Brazil | Valença do Piauí, Piauí |
| **112** | MZUSP_15563 | *M. molossus* | Brazil | Jeremoabo, Bahia |
| **113** | MZUSP_26807 | *M. molossus* | Brazil | Gentio de ouro, Santo Inacio, Bahia |
| **114** | MZUSP_15577 | *M. molossus* | Brazil | Cocorobó, Canudos, Bahia |
| **115** | MZUSP_4089 | *M. molossus* | Brazil | Pinto, Bahia |
| **116** | UFPE 1915 | *M. molossus* | Brazil | Granja, Ceará |
| **117** | UFPE 3076 | *M. molossus* | Brazil | Lagoa Salgada, Rio Grande do Norte |
| **118** | UFPE 3075 | *M. molossus* | Brazil | Lagoa Salgada, Rio Grande do Norte |
| **119** | UFPE 3071 | *M. molossus* | Brazil | Lagoa Salgada, Rio Grande do Norte |
| **120** | UFPE 3068 | *M. molossus* | Brazil | Lagoa Salgada, Rio Grande do Norte |
| **121** | UFPE 3119 | *M. molossus* | Brazil | Araripina, Pernambuco |
| **122** | UFPE 3120 | *M. molossus* | Brazil | Araripina, Pernambuco |
| **123** | UFPE 3118 | *M. molossus* | Brazil | Araripina, Pernambuco |
| **124** | MZUSP_16868 | *M. molossus* | Brazil | Valença do Piauí, Piauí |
| **125** | MZUSP_16866 | *M. molossus* | Brazil | Valença do Piauí, Piauí |
| **126** | MZUSP_16867 | *M. molossus* | Brazil | Valença do Piauí, Piauí |
| **127** | MZUSP_16846 | *M. molossus* | Brazil | Crato, Ceará |
| **128** | MZUSP_16860 | *M. molossus* | Brazil | Crato, Ceará |
| **129** | MZUSP_16815 | *M. molossus* | Brazil | Crato, Ceará |
| **130** | MZUSP_16809 | *M. molossus* | Brazil | Crato, Ceará |
| **131** | MZUSP_16862 | *M. molossus* | Brazil | Crato, Ceará |
| **132** | MZUSP_16836 | *M. molossus* | Brazil | Crato, Ceará |
| **133** | MZUSP_16848 | *M. molossus* | Brazil | Crato, Ceará |
| **134** | MZUSP_16858 | *M. molossus* | Brazil | Crato, Ceará |
| **135** | MZUSP_16847 | *M. molossus* | Brazil | Crato, Ceará |
| **136** | MZUSP_16854 | *M. molossus* | Brazil | Crato, Ceará |
| **137** | MZUSP_16855 | *M. molossus* | Brazil | Crato, Ceará |
| **138** | MZUSP_16856 | *M. molossus* | Brazil | Crato, Ceará |
| **139** | MZUSP_16857 | *M. molossus* | Brazil | Crato, Ceará |
| **140** | MZUSP_16859 | *M. molossus* | Brazil | Crato, Ceará |
| **141** | MZUSP_16833 | *M. molossus* | Brazil | Crato, Ceará |
| **142** | MZUSP_16832 | *M. molossus* | Brazil | Crato, Ceará |
| **143** | MZUSP_16818 | *M. molossus* | Brazil | Exu, Pernambuco |
| **144** | MZUSP_16812 | *M. molossus* | Brazil | Exu, Pernambuco |
| **145** | MZUSP_16805 | *M. molossus* | Brazil | Exu, Pernambuco |
| **146** | MZUSP_16811 | *M. molossus* | Brazil | Exu, Pernambuco |
| **147** | MZUSP_16817 | *M. molossus* | Brazil | Exu, Pernambuco |
| **148** | MZUSP_16792 | *M. molossus* | Brazil | Exu, Pernambuco |
| **149** | MZUSP_16796 | *M. molossus* | Brazil | Exu, Pernambuco |
| **150** | MZUSP_16804 | *M. molossus* | Brazil | Exu, Pernambuco |
| **151** | MZUSP_16821 | *M. molossus* | Brazil | Exu, Pernambuco |
| **152** | MZUSP_16807 | *M. molossus* | Brazil | Exu, Pernambuco |
| **153** | MZUSP_16797 | *M. molossus* | Brazil | Exu, Pernambuco |
| **154** | MZUSP_16798 | *M. molossus* | Brazil | Exu, Pernambuco |
| **155** | UFPB 11633 | *M. molossus* | Brazil | Canindé de São Francisco, Sergipe |
| **156** | UFPB 6185 | *M. molossus* | Brazil | Reserva particular do patrimônio natural (RPPN) fazenda Almas, São José dos Cordeiros, Paraíba |
| **157** | UFPE 2561 | *M. molossus* | Brazil | Granja, Ceará |
| **158** | UFPE 2580 | *M. molossus* | Brazil | Granja, Ceará |
| **159** | UFPE 3073 | *M. molossus* | Brazil | Lagoa Salgada, Rio Grande do Norte |
| **160** | UFPE 3166 | *M. molossus* | Brazil | Araripina, Pernambuco |
| **161** | UFPE 3074 | *M. molossus* | Brazil | Lagoa Salgada, Rio Grande do Norte |
| **162** | UFPE 3070 | *M. molossus* | Brazil | Lagoa Salgada, Rio Grande do Norte |
| **163** | UFPE3077 | *M. molossus* | Brazil | Lagoa Salgada, Rio Grande do Norte |
| **164** | UFPE 3069 | *M. molossus* | Brazil | Lagoa Salgada, Rio Grande do Norte |
| **165** | UFPE 3121 | *M. molossus* | Brazil | Araripina, Pernambuco |
| **166** | MZUSP_8730 | *M. molossus* | Brazil | Pacoti, Ceará |
| **167** | UFPB 8770 | *M. molossus* | Brazil | Mata do Pau Ferro, Areia, Paraíba |
| **168** | MZUSP_4433 | *M. molossus* | Brazil | Eurinepé, Amazônia |
| **169** | MZUSP_371 | *M. molossus* | Brazil | Manaus, Amazônia |
| **170** | MZUSP_4387 | *M. molossus* | Brazil | Caxiricatuba, Pará |
| **171** | MZUSP_4461 | *M. molossus* | Brazil | Caxiricatuba, Pará |
| **172** | MZUSP_4423 | *M. molossus* | Brazil | Caxiricatuba, Pará |
| **173** | MZUSP_4407 | *M. molossus* | Brazil | Caxiricatuba, Pará |
| **174** | MZUSP_4431 | *M. molossus* | Brazil | Caxiricatuba, Pará |
| **175** | MZUSP_23804 | *M. molossus* | Brazil | Caxiricatuba, Pará |
| **176** | MZUSP_4386 | *M. molossus* | Brazil | Caxiricatuba, Pará |
| **177** | MZUSP_15582 | *M. molossus* | Brazil | Boim, Pará |
| **178** | MZUSP_19883 | *M. molossus* | Brazil | Manaus, Amazônia |
| **179** | MZUSP_27896 | *M. molossus* | Brazil | Santa Maria do Boiaçu, Paraná |
| **180** | MZUSP_27900 | *M. molossus* | Brazil | Santa Maria do Boiaçu, Paraná |
| **181** | MZUSP_27894 | *M. molossus* | Brazil | Santa Maria do Boiaçu, Paraná |
| **182** | MZUSP_27895 | *M. molossus* | Brazil | Santa Maria do Boiaçu, Paraná |
| **183** | RRM32 | *M. molossus* | Brazil | Carutapera, Maranhão |
| **184** | RRM43 | *M. molossus* | Brazil | Carutapera, Maranhão |
| **185** | RRM109 | *M. molossus* | Brazil | Godofredo Viana, Maranhão |
| **186** | RRM116 | *M. molossus* | Brazil | Godofredo Viana, Maranhão |
| **187** | MRR04 | *M. molossus* | Brazil | Carutapera, Maranhão |
| **188** | MRR05 | *M. molossus* | Brazil | Carutapera, Maranhão |
| **189** | MRR14 | *M. molossus* | Brazil | Carutapera, Maranhão |
| **190** | MRR18 | *M. molossus* | Brazil | Carutapera, Maranhão |
| **191** | MRR26 | *M. molossus* | Brazil | Carutapera, Maranhão |
| **192** | MRR33 | *M. molossus* | Brazil | Carutapera, Maranhão |
| **193** | MRR37 | *M. molossus* | Brazil | Carutapera, Maranhão |
| **194** | MRR98 | *M. molossus* | Brazil | Cândido Mendes, Maranhão |
| **195** | MRR106 | *M. molossus* | Brazil | Cândido Mendes, Maranhão |
| **196** | MRR108 | *M. molossus* | Brazil | Cândido Mendes, Maranhão |
| **197** | MRR112 | *M. molossus* | Brazil | Cândido Mendes, Maranhão |
| **198** | MRR114 | *M. molossus* | Brazil | Cândido Mendes, Maranhão |
| **199** | MRR115 | *M. molossus* | Brazil | Cândido Mendes, Maranhão |
| **200** | MRR122 | *M. molossus* | Brazil | Cândido Mendes, Maranhão |
| **201** | MRR124 | *M. molossus* | Brazil | Cândido Mendes, Maranhão |
| **202** | MZUSP_4464 | *M. molossus* | Brazil | Eurinepé, Amazônia |
| **203** | MZUSP_4443 | *M. molossus* | Brazil | Eurinepé, Amazônia |
| **204** | MZUSP_4441 | *M. molossus* | Brazil | Caxiricatuba, Pará |
| **205** | MZUSP_4435 | *M. molossus* | Brazil | Caxiricatuba, Pará |
| **206** | MZUSP_4462 | *M. molossus* | Brazil | Caxiricatuba, Pará |
| **207** | MZUSP_4447 | *M. molossus* | Brazil | Caxiricatuba, Pará |
| **208** | MZUSP_36546 | *M. molossus* | Brazil | Santana do Araguaia, Pará |
| **209** | RRM27 | *M. molossus* | Brazil | Carutapera, Maranhão |
| **210** | RRM114 | *M. molossus* | Brazil | Godofredo Viana, Maranhão |
| **211** | MRR23 | *M. molossus* | Brazil | Carutapera, Maranhão |
| **212** | MRR34 | *M. molossus* | Brazil | Carutapera, Maranhão |
| **213** | MRR39 | *M. molossus* | Brazil | Carutapera, Maranhão |
| **214** | MRR99 | *M. molossus* | Brazil | Cândido Mendes, Maranhão |
| **215** | MRR126 | *M. molossus* | Brazil | Cândido Mendes, Maranhão |
| **216** | DZUP/CCMZ2359 | *M. rufus* | Brazil | Frederico Westphalen, Rio Grande do Sul |
| **217** | DZUP/CCMZ963 | *M. rufus* | Brazil | Guaraqueçaba, Paraná |
| **218** | DZUP/CCMZ1215 | *M. rufus* | Brazil | RPPN Monte sinai, Mauá da Serra, Paraná |
| **219** | DZUP/CCMZ791 | *M. rufus* | Brazil | Parque estadual do Turvo, Rio Grande do Sul |
| **220** | DZUP/CCMZ788 | *M. rufus* | Brazil | Parque estadual do Turvo, Rio Grande do Sul |
| **221** | DZUP/CCMZ785 | *M. rufus* | Brazil | Parque estadual do Turvo, Rio Grande do Sul |
| **222** | DZUP/CCMZ2346 | *M. rufus* | Brazil | Linha Castelinho, Frederico Westphalen, Rio Grande do Sul |
| **223** | DZUP/CCMZ2352 | *M. rufus* | Brazil | Linha Castelinho, Frederico Westphalen, Rio Grande do Sul |
| **224** | DZUP/CCMZ2354 | *M. rufus* | Brazil | Linha Castelinho, Frederico Westphalen, Rio Grande do Sul |
| **225** | DZUP/CCMZ2355 | *M. rufus* | Brazil | Linha Castelinho, Frederico Westphalen, Rio Grande do Sul |
| **226** | DZUP/CCMZ2350 | *M. rufus* | Brazil | Linha Castelinho, Frederico Westphalen, Rio Grande do Sul |
| **227** | DZUP/CCMZ2353 | *M. rufus* | Brazil | Linha Castelinho, Frederico Westphalen, Rio Grande do Sul |
| **228** | DZUP/CCMZ782 | *M. rufus* | Brazil | Parque estadual do Turvo, Derrubadas, Rio Grande do Sul |
| **229** | DZUP/CCMZ790 | *M. rufus* | Brazil | Parque estadual do Turvo, Derrubadas, Rio Grande do Sul |
| **230** | DZUP/CCMZ780 | *M. rufus* | Brazil | Parque estadual do Turvo, Derrubadas, Rio Grande do Sul |
| **231** | DZUP/CCMZ787 | *M. rufus* | Brazil | Parque estadual do Turvo, Derrubadas, Rio Grande do Sul |
| **232** | DZUP/CCMZ783 | *M. rufus* | Brazil | Parque estadual do Turvo, Derrubadas, Rio Grande do Sul |
| **233** | DZUP/CCMZ789 | *M. rufus* | Brazil | Parque estadual do Turvo, Derrubadas, Rio Grande do Sul |
| **234** | DZUP/CCMZ797 | *M. rufus* | Brazil | Parque estadual do Turvo, Derrubadas, Rio Grande do Sul |
| **235** | DZUP/CCMZ585 | *M. rufus* | Brazil | Recanto Marista, Doutor Camargo, Paraná |
| **236** | DZUP/CCMZ647 | *M. rufus* | Brazil | Ilha Mutun, Porto Rico, Paraná |
| **237** | DZUP/CCMZ784 | *M. rufus* | Brazil | Parque estadual do Turvo, Derrubadas, Rio Grande do Sul |
| **238** | DZUP/CCMZ2368 | *M. rufus* | Brazil | Porto Rico, Paraná |
| **239** | MZUSP_21085 | *M. rufus* | Brazil | Iguape, São Paulo |
| **240** | MZUSP_21086 | *M. rufus* | Brazil | Iguape, São Paulo |
| **241** | MZUSP_21083 | *M. rufus* | Brazil | Iguape, São Paulo |
| **242** | MZUSP_11550 | *M. rufus* | Brazil | Porto Seguro, Bahia |
| **243** | DG 219 | *M. rufus* | Brazil | Ponte dos Carvalhos, Pernambuco |
| **244** | DG 109 | *M. rufus* | Brazil | Saltinho, R. Formosos, Pernambuco |
| **245** | DZUP/CCMZ962 | *M. rufus* | Brazil | Guaraqueçaba, Paraná |
| **246** | DZUP/CCMZ794 | *M. rufus* | Brazil | Parque estadual do Turvo, Derrubadas, Rio Grande do Sul |
| **247** | DZUP/CCMZ786 | *M. rufus* | Brazil | Parque estadual do Turvo, Derrubadas, Rio Grande do Sul |
| **248** | DZUP/CCMZ2349 | *M. rufus* | Brazil | Linha Castelinho, Frederico Westphalen, Rio Grande do Sul |
| **249** | DZUP/CCMZ795 | *M. rufus* | Brazil | Parque estadual do Turvo, Derrubadas, Rio Grande do Sul |
| **250** | DZUP/CCMZ793 | *M. rufus* | Brazil | Parque estadual do Turvo, Derrubadas, Rio Grande do Sul |
| **251** | DZUP/CCMZ798 | *M. rufus* | Brazil | Parque estadual do Turvo, Derrubadas, Rio Grande do Sul |
| **252** | DZUP/CCMZ646 | *M. rufus* | Brazil | Ilha Mutun, Paraná |
| **253** | DZUP/CCMZ2367 | *M. rufus* | Brazil | Porto Rico, Paraná |
| **254** | MZUSP_1489 | *M. rufus* | Brazil | Rio de Janeiro |
| **255** | MZUSP_6067 | *M. rufus* | Brazil | Santos, São Paulo |
| **256** | DZUP/CCMZ2358 | *M. rufus* | Brazil | Frederico Westphalen, Rio Grande do Sul |
| **257** | DZUP/CCMZ2360 | *M. rufus* | Brazil | Frederico Westphalen, Rio Grande do Sul |
| **258** | DZUP/CCMZ2363 | *M. rufus* | Brazil | Frederico Westphalen, Rio Grande do Sul |
| **259** | DZUP/CCMZ2364 | *M. rufus* | Brazil | Frederico Westphalen, Rio Grande do Sul |
| **260** | DZUP/CCMZ2361 | *M. rufus* | Brazil | Frederico Westphalen, Rio Grande do Sul |
| **261** | DZUP/CCMZ2365 | *M. rufus* | Brazil | Frederico Westphalen, Rio Grande do Sul |
| **262** | MZUSP_1511 | *M. rufus* | Paraguay | Sapucay, Departamento de Paraguari, Paraguay |
| **263** | MZUSP_9729 | *M. rufus* | Brazil | Pacoti, Ceará |
| **264** | UFPB 7452 | *M. rufus* | Brazil | Reserva Biólogica Guaribas, Rio Tinto, Paraíba |
| **265** | UFPB 10569 | *M. rufus* | Brazil | Jardim Botânico Benjamim Maranhão, João Pessoa |
| **266** | MZUSP_4438 | *M. rufus* | Brazil | Caxiricatuba, Rio Tapajós, Pará |
| **267** | MZUSP_4391 | *M. rufus* | Brazil | Itacoatiara, Amazônia |
| **268** | MZUSP_17595 | *M. rufus* | Brazil | Macapá, Rio Maruanum, Ampá |
| **269** | MZUSP_4428 | *M. rufus* | Brazil | Caxiricatuba, Rio Tapajós, Pará |
| **270** | MZUSP_4450 | *M. rufus* | Brazil | Caxiricatuba, Rio Tapajós, Pará |
| **271** | MZUSP_4381 | *M. rufus* | Brazil | Caxiricatuba, Rio Tapajós, Pará |
| **272** | MZUSP_36557 | *M. rufus* | Brazil | Santana do Araguaia, Pará |
| **273** | MZUSP_36553 | *M. rufus* | Brazil | Santana do Araguaia, Pará |
| **274** | MZUSP_36551 | *M. rufus* | Brazil | Santana do Araguaia, Pará |
| **275** | MZUSP_36552 | *M. rufus* | Brazil | Santana do Araguaia, Pará |
| **276** | MZUSP_4437 | *M. rufus* | Brazil | Itacoatiara, Amazônia |
| **277** | MZUSP_5630 | *M. rufus* | Brazil | Caxiricatuba, Rio Tapajós, Pará |
| **278** | CESC057 | *M. rufus* | Brazil | Área de Proteção Ambiental do Inhamum, Maranhão |
| **279** | CESC058 | *M. rufus* | Brazil | Área de Proteção Ambiental do Inhamum, Maranhão |
| **280** | CESC062 | *M. rufus* | Brazil | Área de Proteção Ambiental do Inhamum, Maranhão |
| **281** | CESC064 | *M. rufus* | Brazil | Área de Proteção Ambiental do Inhamum, Maranhão |
| **282** | CESC066 | *M. rufus* | Brazil | Área de Proteção Ambiental do Inhamum, Maranhão |
| **283** | CESC069 | *M. rufus* | Brazil | Área de Proteção Ambiental do Inhamum, Maranhão |
| **284** | CESC071 | *M. rufus* | Brazil | Área de Proteção Ambiental do Inhamum, Maranhão |
| **285** | CESC076 | *M. rufus* | Brazil | Área de Proteção Ambiental do Inhamum, Maranhão |
| **286** | CESC077 | *M. rufus* | Brazil | Área de Proteção Ambiental do Inhamum, Maranhão |
| **287** | CESC080 | *M. rufus* | Brazil | Área de Proteção Ambiental do Inhamum, Maranhão |
| **288** | MAST 07 | *M. rufus* | Brazil | Área de Proteção Ambiental do Inhamum, Maranhão |
| **289** | CUMA 58 | *M. rufus* | Brazil | Codó, Maranhão |
| **290** | CUMA 60 | *M. rufus* | Brazil | Codó, Maranhão |
| **291** | CUMA 63 | *M. rufus* | Brazil | Codó, Maranhão |
| **292** | CUMA 64 | *M. rufus* | Brazil | Codó, Maranhão |
| **293** | CUMA 65 | *M. rufus* | Brazil | Codó, Maranhão |
| **294** | MAST 08 | *M. rufus* | Brazil | Área de Proteção Ambiental do Inhamum, Maranhão |
| **295** | CUMA 41 | *M. rufus* | Brazil | Caxias, Maranhão |
| **296** | CUMA 68 | *M. rufus* | Brazil | Codó, Maranhão |
| **297** | CUMA 69 | *M. rufus* | Brazil | Codó, Maranhão |
| **298** | CUMA 71 | *M. rufus* | Brazil | Codó, Maranhão |
| **299** | CUMA 77 | *M. rufus* | Brazil | Codó, Maranhão |
